# 2′-*O* Methylation of Internal Adenosine by Flavivirus NS5 Methyltransferase

**DOI:** 10.1371/journal.ppat.1002642

**Published:** 2012-04-05

**Authors:** Hongping Dong, David C. Chang, Maggie Ho Chia Hua, Siew Pheng Lim, Yok Hian Chionh, Fabian Hia, Yie Hou Lee, Petra Kukkaro, Shee-Mei Lok, Peter C. Dedon, Pei-Yong Shi

**Affiliations:** 1 Novartis Institute for Tropical Diseases, Singapore; 2 Wadsworth Center, New York State Department of Health, Albany, New York, United States of America; 3 Singapore-MIT Alliance for Research and Technology (SMART) Centre, Singapore; 4 Duke-NUS Graduate Medical School, Singapore; 5 Department of Biological Engineering, Massachusetts Institute of Technology, Cambridge, Massachusetts, United States of America; Purdue University, United States of America

## Abstract

RNA modification plays an important role in modulating host-pathogen interaction. Flavivirus NS5 protein encodes N-7 and 2′-*O* methyltransferase activities that are required for the formation of 5′ type I cap (m^7^GpppAm) of viral RNA genome. Here we reported, for the first time, that flavivirus NS5 has a novel internal RNA methylation activity. Recombinant NS5 proteins of West Nile virus and Dengue virus (serotype 4; DENV-4) specifically methylates polyA, but not polyG, polyC, or polyU, indicating that the methylation occurs at adenosine residue. RNAs with internal adenosines substituted with 2′-*O*-methyladenosines are not active substrates for internal methylation, whereas RNAs with adenosines substituted with N^6^-methyladenosines can be efficiently methylated, suggesting that the internal methylation occurs at the 2′-OH position of adenosine. Mass spectroscopic analysis further demonstrated that the internal methylation product is 2′-*O*-methyladenosine. Importantly, genomic RNA purified from DENV virion contains 2′-*O*-methyladenosine. The 2′-*O* methylation of internal adenosine does not require specific RNA sequence since recombinant methyltransferase of DENV-4 can efficiently methylate RNAs spanning different regions of viral genome, host ribosomal RNAs, and polyA. Structure-based mutagenesis results indicate that K61-D146-K181-E217 tetrad of DENV-4 methyltransferase forms the active site of internal methylation activity; in addition, distinct residues within the methyl donor (*S*-adenosyl-L-methionine) pocket, GTP pocket, and RNA-binding site are critical for the internal methylation activity. Functional analysis using flavivirus replicon and genome-length RNAs showed that internal methylation attenuated viral RNA translation and replication. Polymerase assay revealed that internal 2′-*O*-methyladenosine reduces the efficiency of RNA elongation. Collectively, our results demonstrate that flavivirus NS5 performs 2′-*O* methylation of internal adenosine of viral RNA *in vivo* and host ribosomal RNAs *in vitro*.

## Author Summary

We report that flavivirus NS5 has a novel internal RNA methylation activity. Recombinant proteins of NS5 and its N-terminal methyltransferase domain of West Nile virus and Dengue virus (DENV) specifically methylates polyA, but not polyG, polyC, or polyU. RNAs with internal adenosines substituted with 2′-*O*-methyladenosines are not active substrates for internal methylation, suggesting that the internal methylation occurs at the 2′-OH position of adenosine. Mass spectroscopic analysis confirmed that the internal methylation product is 2′-*O*-methyladenosine. Furthermore, the 2′-*O*-methyladenosine could also be detected in DENV genomic RNA. The 2′-*O* methylation of internal adenosine does not require specific RNA sequence context because the DENV methyltransferase can methylate RNAs spanning different regions of viral genome and host ribosomal RNAs at equal efficiencies. Mutagenesis analysis showed that K61-D146-K181-E217 motif of the DENV methyltransferase forms the active site of internal methylation activity; in addition, distinct residues on the surface of the enzyme are critical for the internal methylation activity. Functional analysis showed that internal methylation attenuated viral RNA translation and replication. Overall, our results demonstrate that flavivirus NS5 performs 2′-*O* methylation of internal adenosine of viral RNA *in vivo* and host ribosomal RNA *in vitro*. Such 2′-*O*-methyladenosine modification may modulate virus-host interaction.

## Introduction

Many members within the *Flavivirus* genus from *Flaviviridae* family are important human pathogens, including the four serotypes of Dengue virus (DENV-1 to -4), yellow fever virus (YFV), West Nile virus (WNV), Japanese encephalitis virus (JEV), and tick-borne encephalitis virus (TBEV). These viruses are naturally transmitted by mosquitoes or ticks, causing global burden and threat to public health [1]. The flaviviral genome is a plus-sense RNA of about 11 kb in length. The 5′ end of the flavivirus genome contains a type I cap, followed by the conserved dinucleotide sequence AG (m^7^GpppAmG). The genomic RNA consists of a 5′ untranslated region (UTR), a single open-reading-frame, and a 3′ UTR. The open-reading-frame encodes a long polyprotein that is processed by viral and host proteases into three structural proteins (capsid [C], premembrane [prM], and envelope [E]) and seven nonstructural proteins (NS1, NS2A, NS2B, NS3, NS4A, NS4B, and NS5) [2]. Structural proteins form viral particles, and participate in virus entry and assembly. Nonstructural proteins function in viral RNA replication [2], evasion of innate immune response [3]–[6], as well as virus assembly [7], [8]. Two flavivirus nonstructural proteins have enzymatic activities. NS3 functions as a viral serine protease (together with NS2B as a cofactor) [9], [10], a NTPase [11], an RNA triphophatase [12], and an RNA helicase [13]. NS5 acts as a methyltransferase (MTase) [14], [15] and an RNA-dependant RNA polymerase (RdRp) [16], [17].

We previously showed that the N-terminal domain of flaviviral NS5 protein posses both N-7 and 2′-*O* methylation activities required for the formation of 5′ RNA cap [15]. The MTase catalyzes the two distinct methylation reactions in a sequential manner, GpppA-RNA→m^7^GpppA-RNA→m^7^GpppAm-RNA. Both reactions use *S*-adenosyl-L-methionine (SAM) as the methyl donor and generate *S*-adenosyl-L-homocysteine (SAH) as a by-product. The order of two sequential methylations is dictated by the fact that the 2′-O methylation reaction prefers the substrate m^7^GpppA-RNA to GpppA-RNA, whereas the N^7^ methylation reaction has no preference between substrates GpppA-RNA and GpppAm-RNA [18]. Biochemical and structural studies indicate that flaviviral MTase catalyses the N^7^ and 2′-*O* methylations through an RNA cap-repositioning mechanism [18], [19]. Functional analysis showed that the N^7^ methylation of flaviviral RNA cap is critical for efficient translation [15], whereas the 2′-*O* methylation functions in subverting innate host antiviral response through escape of IFIT-mediated suppression [20].

Most eukaryotic mRNAs contain co- or post-transcriptional modifications, including the 5′ cap structure, internal bases methylation, splicing of introns, and polyadenylation. N^6^-methyladenosine (m^6^A) represents a major internal modified nucleoside. The m^6^A is found in cellular mRNAs from mammals, plants, insects, and yeast [21]–[26] as well as in some viral RNAs [27]–[29]. The m^6^A modification functions in mRNA processing [25], [30], intracellular transporting, and translation [31]. Besides m^6^A, 2′-*O* methylation of ribose represents another common internal nucleoside modification. The 2′-*O* methylation is found in splicesomal small nuclear RNAs (snRNAs) and ribosomal RNAs [32]. Although the exact function of internal 2′-*O* methylations remains elusive, these modifications are clustered in regions of functional importance, such as regions engaged in RNA-RNA interactions [32]. The distinct chemical properties of 2′-*O* methyl group could modulate RNA structure, thermal stability, biochemical interactions, and other aspects of the modified RNA [33].

Here we report that flavivirus NS5 performs methylation at the 2′-OH position of internal adenosine (Am) of RNA. The 2′-*O* methylation occurs specifically at internal adenosine, not at guanosine, cytidine, or uridine. Mutagenesis analysis indicates that K61-D146-K181-E218 tetrad of the DENV-4 MTase forms the active site to catalyze internal methylation. Functional studies, using flavivirus luciferase replicon and genome-length RNAs, indicate that internal Am modification reduces viral RNA translation and RNA synthesis. Furthermore, we found that recombinant flavivirus NS5 can methylate host ribosomal RNAs *in vitro*.

## Results

### Flavivirus NS5 methylates RNA without a cap structure

**Figure 1:**
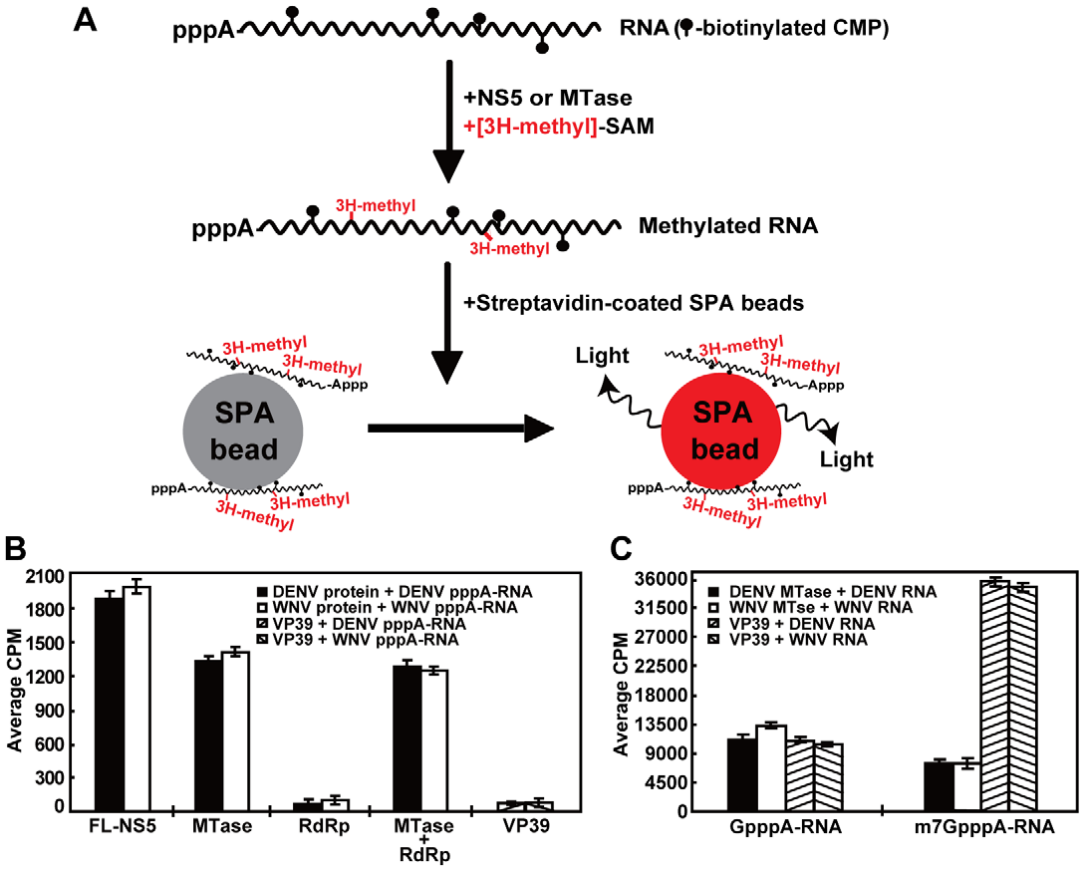
Internal methylation of RNA by flavivirus NS5 and MTase domain. (A) The principle of scintillation proximity assay (SPA). CMP-biotinylated RNA was methylated by enzyme using [^3^H-methyl]-SAM. The biotinylated RNA containing ^3^H-methyl is captured by streptavidin-coated SPA scintillation beads, leading to a signal that can be measured using a MicroBeta counter. (B) SPA analysis of internal methylation of flaviviral RNAs. Uncapped pppA-RNAs, representing the 5′-terminal 190 nt of WNV genome or the 5′-terminal 211 nt of DENV genome, were methylated by indicated recombinant proteins. The combination of protein and pppA-RNA for each reaction is depicted. (C) SPA analysis of RNA cap methylations. GpppA-RNA or m^7^GpppA-RNA, representing the first 190 nt of WNV genome or the first 211 nt of DENV genome, was methylated using the indicated MTases. Average results and standard deviations from three independent experiments are shown.

We developed a scintillation proximity assay (SPA) detect methylation of RNA without a 5′ cap structure (Figure 1A). A pppA-RNA (with 5′ triphosphate) representing the first 211 nt of DENV genome sequence was *in vitro* transcribed in the presence of biotinylated CTP. The biotinylated pppA-RNA was incubated with DENV-4 MTase in the presence of [methyl-^3^H]-SAM. The methylation reaction was then incubated with SPA beads coated with streptavidin. If the RNA is methylated, binding of the biotinylated RNA to the streptavidin SPA beads brings the [methyl-^3^H]-labeling into close proximity to the scintillant (embedded in the beads), leading to a signal that can be measured by a scintillation counter. As shown in Figure 1B, the pppA-RNA gained [methyl-^3^H]-signal upon treatment of DENV-4 MTase. In contrast, no ^3^H-activity was detected after the pppA-RNA was treated with DENV-4 RdRp domain. Addition of the RdRp domain to the MTase domain did not improve the methylation activity, whereas the full-length (FL) NS5 showed higher activity than the MTase domain alone. Interestingly, similar amounts of ^3^H-activity were detected after the WNV pppA-RNA and DENV-4 pppA-RNA were treated with DENV-4 FL NS5 and WNV FL NS5, respectively (data not shown). These results demonstrate that (i) DENV-4 MTase can methylate viral RNA without a 5′ cap structure; (ii) the RdRp domain could enhance the MTase activity, but only when the two domains are physically connected; and (iii) WNV and DENV-4 NS5 can cross methylate heterologous viral RNA.

To exclude the possibility that the observed methylation occurs at the first nucleotide A (where 2′-*O* methylation occurs after the pppA-RNA was capped with a 5′ G [i.e., GpppA-RNA]), we prepared a pppGGA-RNA that contained two extra G residues (underlined) to the 5′ end of authentic viral sequence. Methylation reactions showed that, compared with the pppA-RNA, the addition of two G residues did not change the methylation signals (data not shown). These results suggest that (i) the observed methylation activity is not dependent on the position of the first A residue; (ii) the ^3^H-signals could be derived from internal methylation of the RNAs without 5′ cap.

We expanded the above observation to WNV, another member of flavivirus. Recombinant proteins of WNV FL NS5, MTase domain, RdRp domain were prepared. SPA analysis using pppA-RNA representing the first 190 nt of the WNV genome sequence showed that both FL NS5 and MTase domain, but not RdRp domain, could methylate the pppA-RNA (Figure 1B). As a negative control, vaccinia virus VP39, a known 2′-*O* MTase of RNA cap, did not methylate pppA-RNA containing DENV-4 or WNV sequence (Figure 1B). In contrast, VP39 efficiently methylated m^7^GpppA-RNA (to m^7^GpppAm-RNA) and GpppA-RNA (to GpppAm-RNA); the methylation signals derived from m^7^GpppA-RNA were higher than those derived from GpppA-RNA (Figure 1C), confirming that VP39 prefers methylating RNA cap with the N^7^ position of guanine pre-methylated [34]. As expected, both DENV-4 and WNV MTases could methylate GpppA-RNA and m^7^GpppA-RNA; the signals derived from the former substrate were greater than those derived from the latter substrate (Figure 1C). This is because flavivirus MTase could methylate two positions on substrate GpppA-RNA (to m^7^GpppAm-RNA), whereas it can only methylate one position on substrate m^7^GpppA-RNA (to m^7^GpppAm-RNA). Comparison of methylation signals showed that (i) the VP39-mediated 2′-*O* methylation is more robust than the flavivirus MTase-mediated cap methylations; and (ii) flavivirus MTase methylates RNA cap more efficiently than internal nucleoside. Taken together, these results indicate that flavivirus NS5 can methylate RNA without a cap structure, possibly through methylating internal nucleoside(s).

### Optimal conditions for internal methylation reaction

**Figure 2:**
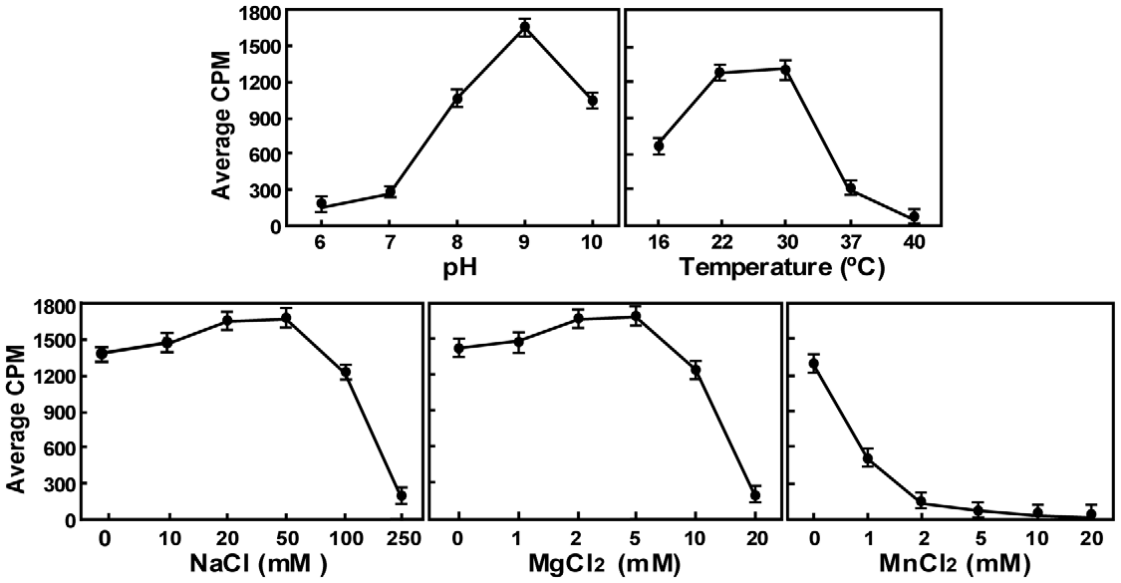
Optimal conditions for internal methylation for DENV-4 NS5 MTase. SPA-based methylation assays were performed using uncapped pppA-RNA substrate (representing the first 211 nt of DENV genome). The reaction mixtures were incubated for 1 h at room temperature. Optimal pH, temperature, NaCl concentration, MgCl_2_ concentration, and MnCl_2_ concentration were obtained by titrating individual parameter while keeping other parameters at the optimal levels. Average results and standard deviations were obtained from three independent experiments.

Using substrate pppA-RNA (5′ 211 nt of DENV genomic RNA) and DENV-4 MTase, we determined the optimal condition for internal methylation activity. As shown in Figure 2, the activity reached maximum when performed at 22°C to 30°C in pH 9.0 buffer containing 50 mM NaCl and 5 mM MgCl_2_. Addition of MnCl_2_ inhibited the internal methylation activity.

### Specific methylation of adenosine

**Figure 3:**
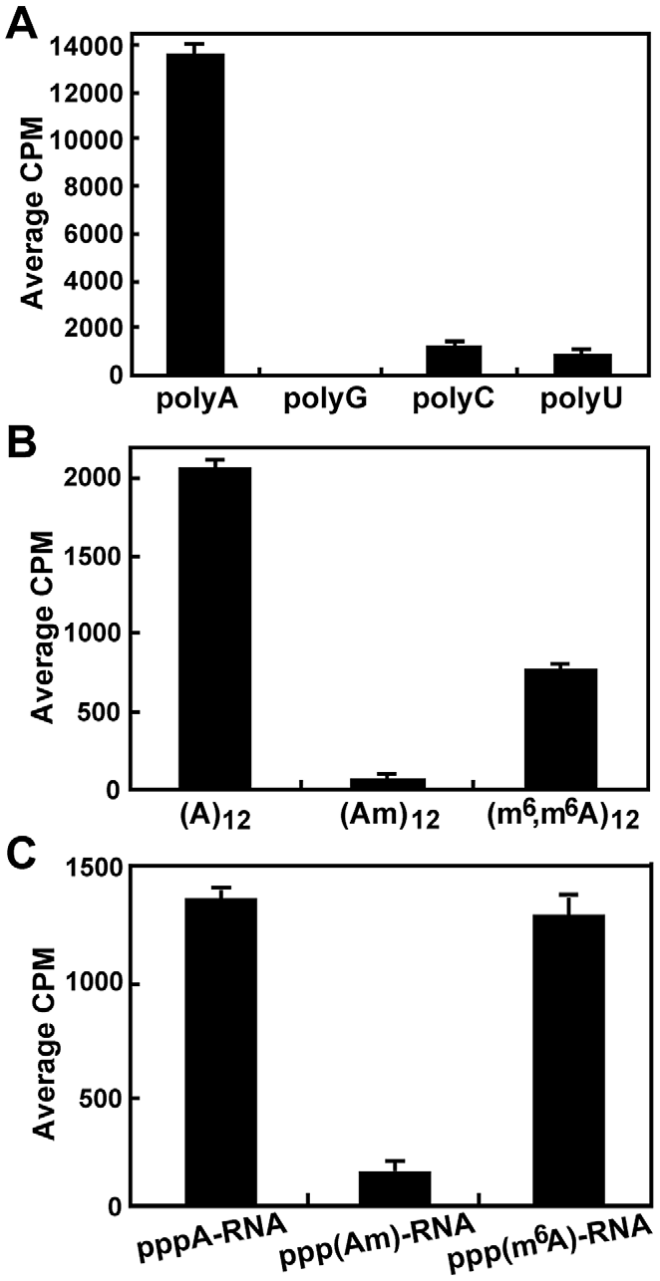
2′-*O* methylation of internal adenosine. (A) Incorporation of ^3^H-methyl into polyA. Homopolymer RNAs (1 µg) were incubated with 2 µg of DENV-4 MTase in the presence of [^3^H-methyl]-SAM. After the methylation reaction, the un-incorporated [^3^H-methyl]-SAM was removed by RNeasy kit. The amount of ^3^H-methyl incorporation was measured by a MicroBeta counting. (B) SPA-based methylation analysis of oligo (A)_12_, (Am)_12_, and (m^6^,m^6^A)_12_. All three RNA oligos were 3′-end biotinylated to facilitate SPA analysis. Am indicates that the 2′-OH of adenosine is methylated. m^6^,m^6^A indicates that the amino N^6^ position of adenosine is double methylated. (C) SPA-based methylation analysis of DENV-1 RNA. pppA-RNAs, representing the 5′ 211 nt of DENV-1 genome, were *in vitro* transcribed using biotinylated-CTP plus unmodified ATP, 2′-*O*-methyladenosine triphosphate (AmTP), or N^6^ methyl adenosine triphosphate (m^6^ATP). The transcription reactions generated pppA-RNA, ppp(Am)-RNA, and ppp(m^6^A)RNA, respectively. The RNAs were then subjected to SPA-based internal methylation analysis. Average results and standard deviations from three independent experiments are presented.

To identify which nucleoside is internally methylated by DENV-4 MTase, we performed methylation reactions using homopolymer RNAs (polyA, polyG, polyC, or polyU). Since the homopolymer RNAs were not biotinylated, the methylation reactions were purified through an RNeasy column (Qiagen) to remove the un-incorporated [methyl-^3^H]-SAM. The purified RNAs were then measured for the level of ^3^H-methyl incorporation using a scintilation counter. The results showed that DENV-4 MTase efficiently methylated polyA (Figure 3A). No ^3^H-methyl incorporation was detected with polyG, and the incorporations with polyC and polyU were approximately 30-fold less efficient than that of polyA (Figure 3A). The results indicate that (i) DENV-4 MTase preferentially methylates adenosine; (ii) the internal methylation activity does not require a viral RNA sequence.

### 2′-*O* methylation of adenosine

To explore which position of adenosine is methylated, we synthesized three (A)_12_ RNA derivatives, each of which was 3′ terminally biotinylated. Oligo (A)_12_ contained unmodified adenosines; oligo (Am)_12_ contained adenosine with ribose 2′-OH position methylated; and oligo (m^6^,m^6^A)_12_ contained adenosine with adenine N^6^ position double methylated. SPA-based methylation assays showed that oligo (A)_12_ was an active substrate for DENV-4 MTase (Figure 3B). In contrast, no methylation activity was observed for oligo (Am)_12_, while oligo (m^6^,m^6^A)_12_ had a 53% reduction of the methylation activity than that of oligo (A)_12_ (Figure 3B). These results argue that the methylation occurs at the ribose 2′-OH position of adenosine. The reduction of methylation activity of oligo (m^6^,m^6^A)_12_ could be due to steric hindrance between the double N^6^ methyl groups of (m^6^,m^6^A)_12_ and MTase during the methylation reaction.

Next, we introduced 2′-*O*-methyladenine or N^6^ methyl adenine (m^6^A) into DENV-4 pppA-RNA (representing the 5′ 211 nt of DENV-4 genome). The pppA-RNA was *in vitro* transcribed using 2′-*O*-methyladenine triphosphate (AmTP) or N^6^ methyl adenine triphosphate (m^6^ATP) in the absence of unmodified ATP. SPA-based methylation assays showed that the unmodified pppA-RNA and the (m^6^A)-modified pppA-RNA yielded similar levels of methylation signals (Figure 3C). In contrast, only background methylation signal was observed when using the (Am)-modified pppA-RNA. The results again indicate that DENV-4 MTase methylates the ribose 2′-OH position of adenine.

### Identification of 2′-*O*-methyladenosine as the methylation product

**Figure 4:**
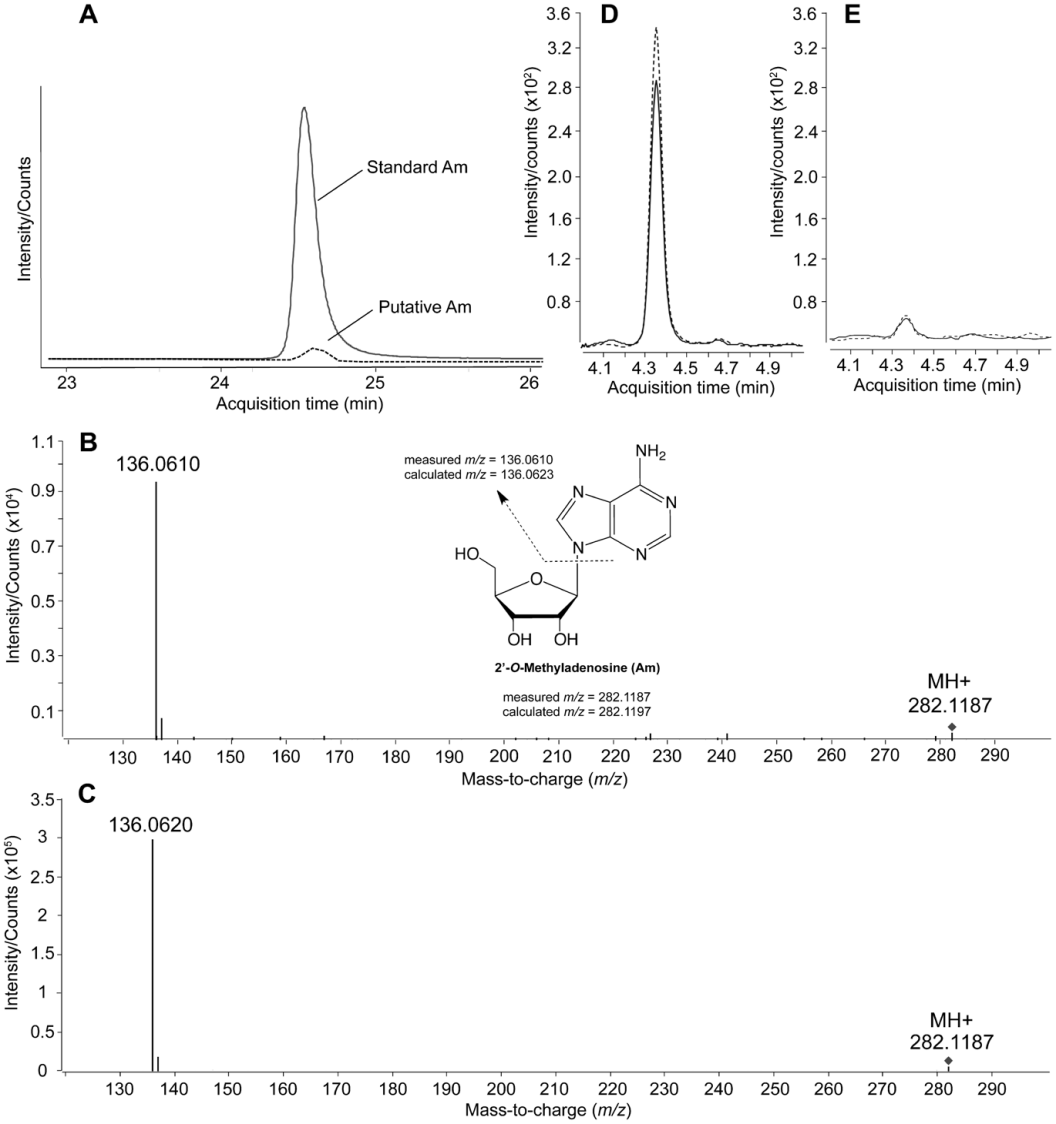
Mass spectrometric analysis of Am in MTase-treated polyA and in DENV genomic RNA. (A) Extracted ion chromatogram of ions with *m/z* 282.1187 from the LC-QTOF scan of putative Am in hydrolysates of DENV-4 MTase-treated polyA and of standard Am. (B,C) CID spectra of the parent ion *m/z* 282.1187 representing standard Am (B) and putative Am in hydrolysates of DENV-4 MTase treated polyA species (C). The inset shows the assignment of structures for the CID spectra. (D,E) LC-MS/MS quantification of Am in WT DENV-1 genomic RNA (D) and MTase E217A mutant DENV-1 genomic RNA (E); the solid and dashed lines represent technical replicates. The different retention time for Am in panel A (∼24.5 min) compared to panels D and E (∼4.4 min) is the result of different HPLC flow rates used for the two studies.

Rigorous chemical identification of Am was achieved by mass spectrometry. High mass-accuracy LC-QTOF analysis of the hydrolysate of DENV-4 MTase-treated polyA revealed only the canonical ribonucleosides (data not shown) and a signal with *m/z* 282.1187, as shown in the extracted ion chromatogram in Figure 4A. This *m/z* value yields a molecular formula of C_11_O_4_N_5_H_15_, which corresponds to a methylated adenosine species. Subsequent analysis by collision-induced dissociation (CID) revealed fragmentation of *m/z* 282.1187 to an ion with *m/z* 136.0620 (Figure 4C), which corresponds to adenine base, with loss of a 2′-*O*-methyl ribose moiety. To confirm that the unknown species was 2′-*O*-methyladenosine (Am), the LC-QTOF analysis with CID was repeated with synthetic Am, which yielded the same HPLC retention time (Figure 4A), *m/z* value, and CID fragmentation pattern as the unknown compound (Figure 4B). Analysis of the RNA for other methylated adenosine (e.g., m^1^A, m^6^A, m^6^_2_A, t^6^A, i^6^A) by direct analysis or comparison to chemical standards yielded no detectable signals. These data demonstrate that the methylation catalyzed by the DENV-4 in polyA is specific for the 2′-OH position of adenosine.

### No sequence preference for internal adenosine methylation

**Figure 5:**
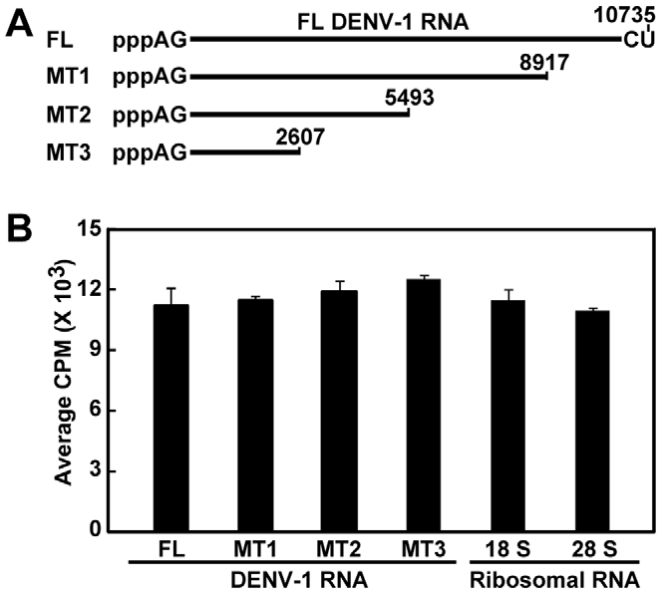
Comparison of internal methylation efficiencies between DENV RNAs and host ribosomal RNAs. (A) Full-length (FL) and 3′ truncated RNAs of DENV-1. pppAG-RNAs, representing the FL and a set of 3′ terminally truncated DENV-1 RNAs, were *in vitro* synthesized. Numbers indicate nucleoside positions of DENV-1 genome (GenBank accession number U88535). (B) Internal methylation analysis. An equal mass (0.5 µg) of FL and truncated DENV-1 RNAs, and human ribosomal 18 S and 28 S RNAs was treated with DENV MTase in the presence of [^3^H-methyl]-SAM. The reactions were purified through an RNeasy column to remove unincorporated [^3^H-methyl]-SAM. The purified RNAs were quantified for internal methylation by a MicroBeta counter. Average results from three experiments are shown; error bars represent standard deviations.

We examined whether flavivirus MTase has sequence preference for internal adenosine methylation. A set of 3′ truncated DENV-1 RNAs were *in vitro* transcribed; each RNA contained a 5′ pppAG sequence without a cap structure (Figure 5A). Equal amounts of FL and truncated viral RNAs (0.5 µg) were treated with DENV-4 MTase in the presence of [^3^H-methyl]-SAM. As shown in Figure 5B, no significant difference in methylation signals was observed between the FL and truncated RNAs, indicating that the MTase does not have sequence preference within viral genome for internal adenosine methylation. This conclusion was further supported by the results that (i) recombinant DENV MTase and WNV MTase could internally methylate WNV RNA and DENV RNA, respectively, at a similar efficiency (data not shown); (ii) cellular ribosomal 18 S and 28 S RNAs were equally methylated by the DENV-4 MTase (Figure 5B). Quantitative LC-MS/MS analysis revealed the MTase-induced increases in Am levels in 18 S and 28 S rRNA of 67.9% and 16.4%, respectively.

### K61-D146-K182-E217 tetrad forms the active site for internal methylation

**Figure 6:**
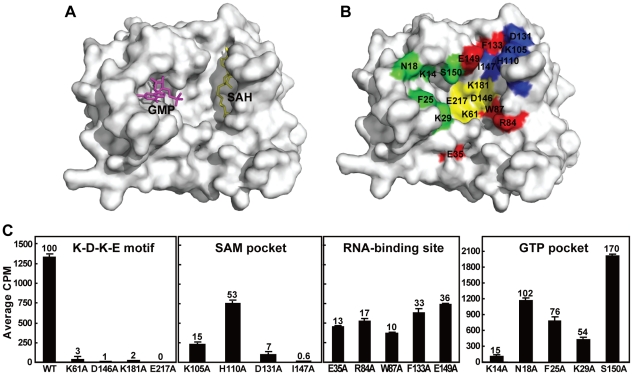
Mutagenesis analysis of DENV-4 MTase. (A) Co-crystal structure of DENV MTase showing SAH (yellow stick) and GMP (pink stick). (B) Surface presentation of DENV MTase depicting mutated amino acids. Mutated residues in the K-D-K-E motif, SAM-binding pocket, RNA-binding site, and GMP-binding pocket are shown in yellow, blue, red, and green, respectively. The images were produced using DENV-2 MTase structure (PDB code: 1L9K) [14] and PyMOL. (C) Effects of mutations of DENV-4 MTase on internal methylation. Biotinylated pppA-RNA (representing the first 211 nt of DENV genomic RNA) was incubated with WT or various mutant MTases in the presence of [^3^H-methyl]-SAM. The reactions were quantified for [^3^H-methyl]-incorporation using SPA analysis. The methylation efficiencies of mutant MTases were compared with that of the WT MTase (set at 100%). Averages of three independent experiments are shown. Error bars indicate standard deviations.

We performed a structure-based mutagenesis analysis of the DENV-4 MTase to identify amino acids that are critical for internal methylation. Crystal structures of flavivirus MTases share three conserved structural elements (Figure 6A): a SAH-binding pocket, a GTP-binding pocket, and a RNA-binding site [14], [35]. For every structural pocket, we prepared a panel of mutant DENV-4 MTases, each containing an Ala substitution of one amino acid (Figure 6B). In addition, Ala substitution was also performed on the K-D-K-E motif, the active site for the 2′-*O* cap methylation [15]. All mutant MTases were analyzed using a DENV pppA-RNA (representing the first 211 nt of genomic RNA) in a SPA-based methylation assay.

Figure 6C summarizes the internal methylation activities of 18 mutant MTases of DENV-4. (i) For the K61-D146-K181-E217 motif (Figure 6B, residues in yellow), Ala substitution of each residue within the tetrad abolished the methylation activity (Figure 6C), suggesting that the K-D-K-E motif forms the active site for internal methylation. (ii) For the SAM-binding pocket (Figure 6B, residues in blue), mutations of K105, D131, and I147 reduced the methylation activity to <20% of the WT activity, whereas mutation of H110 reduced the methylation activity by about 47% (Figure 6C). These results indicate that the SAM pocket is critical for internal methylation by positioning the methyl donor SAM. (iii) For RNA-binding site, each of the five mutations within the RNA-binding site (Figure 6B, residues in red) reduced the activity by >60% (Figure 6C), indicating the importance of these residues in internal methylation activity. (iv) For the GTP-binding pocket (Figure 6B, residues in green), only one (K14) of the five mutations reduced the activity by >60%. Interestingly, S150A mutant increased the activity by 70% (Figure 6C). It is currently not known how the residues within the RNA-binding site and GTP-binding pocket contribute to the methylation activity. Nevertheless, the mutagenesis results indicate that distinct amino acids of the DENV-4 MTase are required for internal methylation activity.

### 2′-*O* methylation of internal adenosine reduces viral translation and RNA synthesis

**Figure 7:**
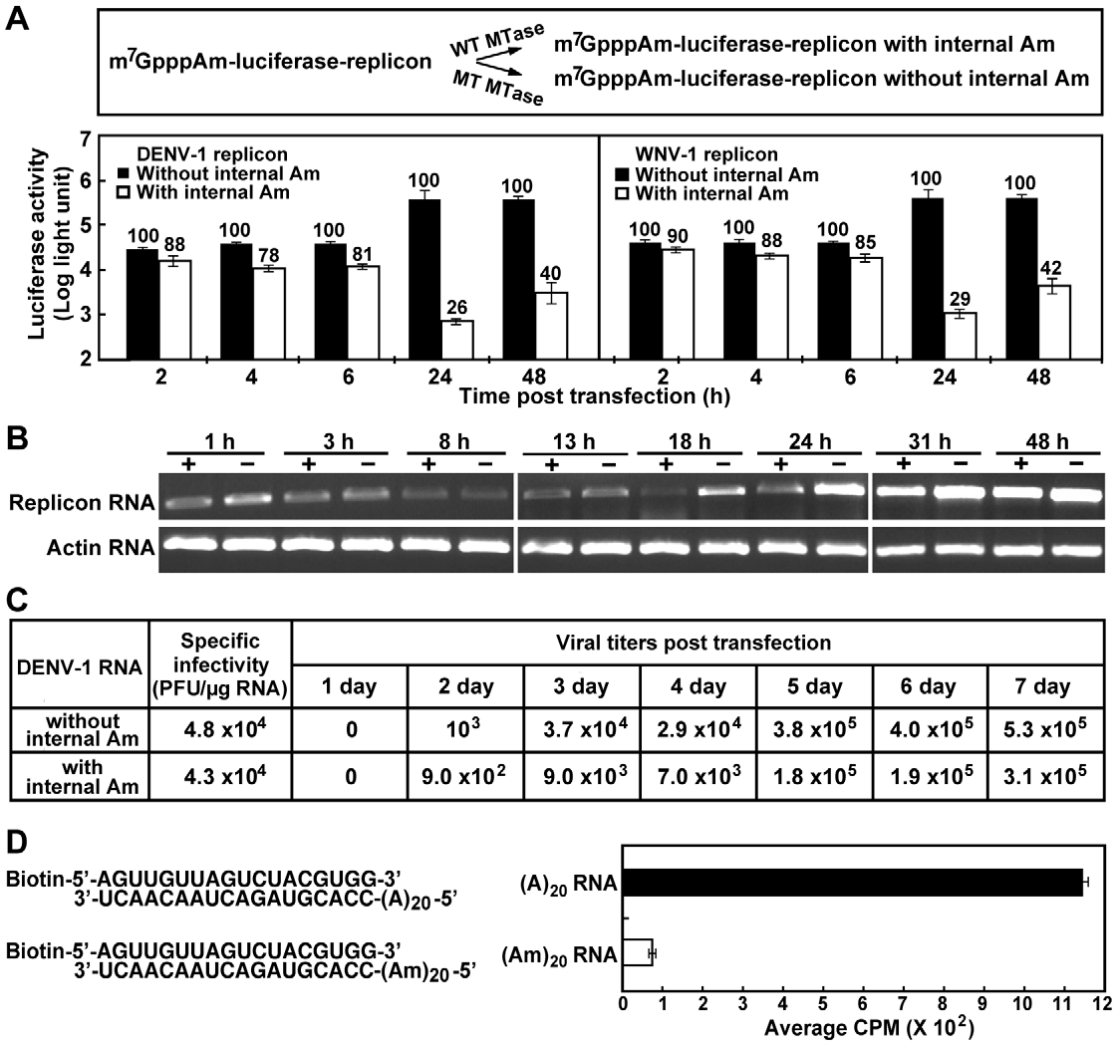
Effects of internal methylation on flavivirus RNA translation and replication. (A) Replicon analysis. Top panel depicts the procedures to prepare replicon RNAs with and without internal adenosine methylations. Bottom panel shows the effects of internal Am modification on viral RNA translation and synthesis. Both DENV-1 and WNV luciferase replicons were used in the analysis. Specifically, equal amounts (2 µg) of replicon RNAs with and without internal Am modifications were electroporated into BHK-21 cells. The transfected cells were assayed for luciferase activities at indicated time points. For each time point, relative luciferase activities were compared between the replicons with internal Am and the replicon without internal Am (set at 100%). Average results and standard deviations from three experiments are presented. (B) RT-PCR analysis. The transfected cells described in (A) were extracted for total cellular RNA at indicated time points. Equal amounts of total cellular RNA (3 µg) were subjected to RT-PCR quantification using primers targeting viral NS5 gene. actin, a host housekeeping gene, was included as a control. The RT-PCR products were analyzed on a 1% agarose gel. One of the three representative experimental results is presented. (C) Effects of internal Am modification on the replication of genome-length RNA. DENV-1 genome-length RNAs with or without internal Am modifications were prepared as depicted in (A). Equal amounts of RNAs with or without internal Am modifications were transfected into BHK-21 cells, and compared for their specific infectivities and virus yields at indicated time points post transfection. (D) Effect of 2′-*O*-methylation on viral polymerase activity. An RNA elongation assay was used to compare the RdRp activities between RNA templates with and without 2′-*O*-methyladenosine. RNA sequences of primer/template are shown (left panel). Incorporation of ^3^H-UTP in to the biotinylated RNA primer in the presence of DENV-4 NS5 was measured (right panel). Average results and standard deviations from three independent experiments are shown.

DNEV-1 and WNV replicons expressing *Renilla* luciferase (RlucRep) were used to analyze the role of internal methylation in viral translation and RNA replication. Transfection of BHK-21 cells with flavivirus RlucRep RNA was previously shown to yield two distinctive peaks, one at 2 to 6 h and another at ≥24 h post-transfection (p.t.). The two luciferase peaks represent viral translation of input RNA and RNA translation of newly synthesized RNA, respectively [36]. As shown in Figure 7A (top panel), DENV-1 and WNV replicon RNAs (containing the 5′ m^7^GppAm cap) were treated with SAM and cognate MTase, resulting in internally methylated RNAs. As a control, the replicon RNAs were treated with cognate E217A MTase (a mutant that is inactive in internal methylation). Equal amounts of the treated replicon RNAs were electroporated into BHK-21 cells. The transfected cells were assayed for luciferase activities at various time points after electroporation. For both DENV-1 and WNV replicon, the WT MTase-treated replicon generated 10–22% less luciferase activity than the mutant MTase-treated replicon at 2 to 6 h p.t. (Figure 7A), suggesting that internal methylation slightly reduces the translation of viral RNA. At 24 and 48 h p.t., the luciferase signals derived from the WT MTase-treated replicons were about 26–42% of the luciferase signals derived from the mutant MTase-treated replicons, suggesting that internal methylation suppresses viral RNA synthesis.

To exclude the possibility that the observed difference in luciferase activity was caused by a difference in transfection efficiency between the replicons with and without internal methylation, we used RT-PCR to quantify the intracellular levels of viral RNA at various time points post-transfection. During the first 13 h p.t., similar levels of viral RNAs were detected between the cells transfected with the WT and mutant MTase-treated RNAs (Figure 7B). This result indicates that (i) the RNA transfection efficiencies were comparable; (ii) internal methylation does not change the stability of the transfected RNA. From 18 to 24 h p.t., the RT-PCR products derived from the WT MTase-treated replicon was much less than those derived from the mutant MTase-treated replicon; this difference became less dramatic from 31 to 48 h p.t. (Figure 7B). The replication difference observed at 18 to 24 h p.t. was most likely due to the difference in internal methylation of the input replicon RNAs. Since both WT and mutant MTase-treated replicons contained WT NS5 gene, the intracelluarly translated WT NS5 protein would methylate progeny viral RNA (and possibly also the transfected replicon RNAs), resulting in less difference in RNA replication observed at 31 to 48 h p.t. (Figure 7B).

Next, we analyzed the effect of internal methylation of genome-length RNA on virus production. Genome-length RNA of DENV-1 was *in vitro* transcribed from an infectious cDNA clone, 5′ capped with m^7^GpppAm (using vaccinia virus capping enzymes and VP39 MTase), and treated with DENV WT or mutant E217A MTase. Transfection of BHK-21 cells with equal amounts of MTase-treated genome-length RNAs showed similar values of specific infectivity, 4.3×10^4^ and 4.8×10^4^ PFU per µg of transfected RNA, respectively (Figure 7C). However, cells transfected with the WT MTase-treated RNA produced slightly less virus than the cells transfected with the mutant MTase-treated RNA (Figure 7C), suggesting that internal methylation could attenuate virus production. We performed genome-length sequencing of the viruses recovered on day 7 post-infection. No adaptive mutation was detected. The results suggest that the decrease in difference of viral titers at later time points between the WT and mutant MTase-treated RNAs is due to a simple dilution of the un-methylated transfected RNA with methylated progeny RNAs produced in subsequent rounds of replication and re-infection.

### Effect of 2′-*O*-methylation on viral polymerase activity

A SPA-based RNA elongation assay was established to compare the viral RdRp activities between RNA templates with and without 2′-*O*-methyladenosine. As shown in Figure 7D (left panel), a 5′ terminally biotinylated RNA oligo was annealed to a template (A)_20_ or (Am)_20_. Incorporation of ^3^H-labelled UTP into the biotinylated RNA in the presence of recombinant DENV-4 NS5 was measured. The amount of ^3^H-UMP incorporation into the (Am)_20_ template was about 16-fold less than that into the (A)_20_ template (Figure 7D, right panel). A similar result was observed when RNA elongation activity was monitored by incorporation of ^32^P-UMP and the RNA products were analyzed on a denaturing polyacrylamide gel (data not shown). Collectively, the results indicate that 2′-*O*-methyladenosine in RNA template reduces the efficiency of RNA elongation.

### DENV genomic RNA contains 2′-*O*-methyladenosine at sites internal to the 5′-cap

To demonstrate the biological relevance of internal adenosine methylation, we purified WT and MTase E217A mutant DENV-1 virions. Viral genomic RNAs were extracted from the virions and enzymatically digested to ribonucleoside form, followed by LC-MS analysis of the ribonucleosides (see Materials and Methods for details). The analyses revealed that genomic RNA extracted from the WT virion contained Am at a frequency of 3.4±0.05 Am per genome (Figure 4D), while genomic RNA purified from the MTase mutant virion did not contain significant levels of Am (Figure 4E). The results clearly demonstrate that internal 2′-*O*-methyladenosine exists in DENV genomic RNA, though at a low frequency.

## Discussion

The current study has provided four lines of evidence to demonstrate that flavivirus MTase performs internal 2′-*O* methylation of adenine (Am). (i) Recombinant NS5 of DENV-4 and WNV can methylate viral RNA without a 5′ cap structure. Recombinant NS5 of other DENV serotypes (DENV-2 and -3) can also perform internal methylation (data not shown). (ii) DENV-4 MTase methylates polyA, but not polyG, polyC, or polyU. This is in stark contrast to flavivirus N-7 and 2′-*O* cap methylations which require RNA substrates with distinct viral sequence and structural elements [19], [37]. This is also different from the requirement of cellular mRNA m^6^A methylation, which occurs only within the GAC or AAC sequences (where A is methylated) [28], [38]. (iii) RNAs containing Am are not active substrates for internal methylation, whereas RNAs containing m^6^A are active substrates for internal methylation. (iv) Mass spectrometric analysis showed that the methylated product was 2′-*O*-methyladenosine. Importantly, we showed that genomic RNA extracted from DENV virion contains internal 2′-O-methyladenine albeit at low frequency. It should be noted that the internal adenosine methylation activity of flavivirus MTase is much lower than the N-7 and 2′-*O* cap methylations (compare Figures 1B and 1C). The observed internal methylation activity seems unique to flavivirus MTase since vaccinia virus VP39, a well known 2′-*O* MTase of RNA cap, did not show any internal methylation activity.

Flavivirus internal Am modification exhibits a number of properties similar to that of 2′-*O* cap methylation. (i) Both methylations occur at the ribose 2′-OH position of adenosine (i.e., m^7^GpppApN→m^7^GpppAmpN and NpApN→NpAmpN). For 2′-*O* cap methylation, we previously showed that substitution of the wild-type m^7^GpppA with m^7^GpppG completely abolished the 2′-*O* cap methylation of WNV RNA [19]. For 2′-*O* internal methylation, DENV MTase does not seem to have preference for RNA sequence context within viral genome; it can even methylate host ribosomal RNAs at an equal efficiency (Figure 5). (ii) Both methylations transfer a methyl group from SAM molecule that is bound to the same pocket of the enzyme. This is supported by two evidences: only one SAM-binding site is observed in flavivirus MTase crystal structure; mutations of the SAM-binding pocket abolished both cap methylations [18], [37] as well as internal adenosine methylation (Figure 4C). (iii) Both activities use the K61-D146-K181-E217 tetrad as an active site. Ala-substitution of each of the tetrad lead to complete loss of 2′-*O* cap methylation [18], [37] and internal adenosine methylation (Figure 4C). (iv) Both reactions require a similar optimal buffer conditions (e.g., optimal pH at 9.0) [35], [37]. These similarities suggest that the two reactions share a common mechanism of catalysis. We recently solved the co-crystal structure of DENV-3 MTase in complex with SAH and an m^7^GpppA-RNA oligo (Lescar et al., submitted for publication). The co-crystal structure supports a mechanism that, during 2′-*O* cap methylation or internal adenosine methylation, K181 is deprotonated, leading to the deprotonation of the 2′-OH of ribose. The deprotonated 2′-OH of ribose then interacts with the CH_3_ group from SAM molecule, resulting in the formation of the S_N_-2-like transition state to accomplish the methyl transfer. A similar mechanism has previously proposed for vaccinia VP39 and other MTases [39], [40].

We explored the function of internal Am by analyzing its effects on viral RNA translation and replication. Using luciferase replicons of DENV-1 and WNV treated with respective recombinant MTases, we found that internal Am reduced the efficiency of RNA translation by approximately 10–22% (Figure 7A). This is in contrast to the observation that m^6^A modification enhances mRNA translation [31]. It is currently not clear how the two distinct methylations of adenosine modulate translation with opposite outcomes. For RNA replication, we found that internal Am significantly reduced viral RNA synthesis of DENV-1 replicon (indicated by luciferase reporting signals; Figures 7B and 7C) as well as the replication of genome-length RNA (Figure 7C). The lower efficiency of RNA synthesis could result from a decrease in input RNA translation. Alternatively, the internal Am could directly attenuate RNA replication during the first round of viral replication. The latter explanation was supported by the biochemical results showing that 2′-*O*-methyladenosine in RNA template reduces the efficiency of NS5-mediated RNA elongation (Figure 7D). In addition, since the 2′-*O* methylation of viral RNA cap functions in subverting innate host antiviral response [20], [41], it is possible that internal methylation of viral RNA could also modulate virus-host interactions. It should be noted that because the same K-D-K-E active site of MTase is responsible for 2′-O methylations of both 5′ RNA cap and internal adenosine, the observed evasion of immune response could be due to lack of methylation(s) of RNA cap and/or internal adenosine. Indeed, we found that 2′-O MTase mutant virus triggered stronger innate immune response than the WT virus did in cell culture (manuscript in preparation). It is currently impossible to differentiate the effect of the two types of methylations (5′ RNA cap and internal adenosine) on evasion of host immune response. In eukaryotes, 2′-*O* methylation is abundant in splicesomal snRNA and ribosomal RNA; however, 2′-*O* methylation has not been reported for mRNA. In spliceosomal snRNA, 2′-*O* methylation occurs at the branch point adenosine; such modification was shown to block pre-mRNA splicing in *Xenopus oocytes* [42], [43]. In ribosomal RNA, 2′-*O* methylation could increase the stability of RNA conformation [32].

We showed that flavivirus MTase can methylate cellular ribosomal RNAs *in vitro* (Figure 5B). This observation raises the possibility that the viral MTase may modulate host RNAs in infected cells. During flavivirus infection, only a small portion of expressed NS5 protein is located within the replication complex; majority of the viral NS5 protein is dispersed outside the replication site [44]. DENV NS5 translocates into nucleus in infected cells, and the distribution of DENV NS5 between cytoplasm and nucleus is regulated by the phosphorylation status of the NS5 protein [44]. Future studies are needed to examine whether host RNAs are modified by flavivirus MTase in infected cells.

## Materials and Methods

### Chemicals and reagents

All chemicals and reagents were of the highest purity available and were used without further purification. Nuclease P1 and phosphodiesterase I were purchased from USB (Cleveland, OH, USA). Coformycin were obtained from the National Cancer Institute Open Chemical Repository (Bethesda, MD USA). Deferoxamine mesylate, tetrahydrouridine, butylated hydroxytoluene, alkaline phosphatase, and 2′-*O*-methyladenosine were purchased from Sigma Chemical Co. (St. Louis, MO, USA). Thermo Hypersil aQ HPLC column was purchased from Thermo Fisher Scientific (Waltham, MA, USA). Experiments were performed on an Agilent LC/QTOF 6520 system (Santa Clara, CA, USA).

### RNA substrates

Two types of RNA substrates were prepared for methylation analysis. Type one RNA contains non–viral sequence. Synthetic RNA oligos include (A)_12_, (Am)_12_ (“m” indicates 2′-*O*-methyl adenosine), and (m^6^_2_A)_12_ (dimethylation of the exocyclic N^6^-position of adenosine). The 3′ end of each oligo is biotinylated. These oligos were synthesized by Dhamacon, Inc (Lafayette, CO). In addition, polyA, polyC, polyG, and polyU without any biotinylation were also used in the methylation assay. These homopolymers were purchased from Sigma Aldrich. For the 18S and 28S rRNA species, total RNA was isolated from CCRF-SB B-lymphocytic leukemia cells (ATCC, Manassas, VA, USA) by homogenizing cells (repetitive pipetting) in 1 ml of Trizol reagent followed by extraction with 0.2 ml volume of chloroform and isopropanol precipitation of the aqueous phase. The 18 S and 28 S rRNA species were purified by size-exclusion HPLC using an Agilent 1200 HPLC system (Agilent Technologies, Santa Clara, CA, USA) equipped with an Agilent Bio-SEC5 column (2000 Å, 300 mm×7.8 mm) eluted at 60°C with 100 mM ammonium acetate at 0.5 ml/min. Collected fractions were desalted using Ambion Millipore 10K MWCO columns (Millipore, Billerica, MA, USA) and the quality and concentration of the resulting rRNAs was assessed by analysis on an Agilent Bioanalyzer (Agilent Technologies, Santa Clara, CA, USA; RNA 6000 Nano Kit).

Type two RNA contains viral sequence. RNAs representing the first 190 nucleotides (nt) of WNV genome or the first 211 nt of DENV-1 genome were *in vitro* transcribed from PCR-generated DNA templates as reported previously [15], [37]. The *in vitro* transcription was performed using MEGAshortscription Kit (Applied Biosystems). Biotinylated RNAs were produced using biotinylated-CTP and regular CTP at a ratio of 1∶2. RNAs containing 2′-*O*-methyladenosines or N^6^-methyladenosines (m^6^A) were *in vitro* transcribed using 2′-*O*-methyladenosine triphosphate (AmTP) or N^6^-methyladenosine triphosphate (m^6^ATP) in the absence of unmodified ATP. RNAs with 5′ m^7^GpppA or GpppA cap were prepared by incubation of *in vitro* transcribed pppA-RNA with vaccinia virus capping enzyme (Epicetre) in the presence of GTP with or without SAM, respectively. RNA with 5′ m^7^GpppAm cap was prepared by vaccinia capping enzyme and VP39 2′-*O* MTase following the manufacturer's protocol (Epicetre). All RNA transcripts were purified twice through Sephadex G-25 spin columns (GE Healthcare), extracted with phenol-chloroform, precipitated with ethanol, and resuspended in RNase-free water.

### Analysis of internal RNA methylation *in vitro*

Three assays were performed to detect internal RNA methylation. The first assay used SPA format in a 96-well plate (Figure 1A). Biotinylated RNA species (6 pmol) were incubated with 18 pmol of full-length NS5 (or MTase domain) and 1 µCi of [^3^H-methyl]-SAM (PerkinElmer) in buffer containing 50 mM Tris-HCl (pH 9.0) and 50 mM NaCl at room temperature for 1 h. The reactions were terminated by addition of an equal volume of 2× stop solution (containing 100 mg of SPA beads coated with streptavidin in 50 mM Tris-HCl [pH 7.0], 40 mM EDTA, and 150 mM NaCl). The 96-well plate was agitated at room temperature for 15 min, and measured for ^3^H-methyl incorporation (into RNA) by a MicroBeta counter (Perkin-Elmer). The full-length NS5 and MTase domain from both WNV and DENV-4 were used in the methylation assays. The MTase domains of WNV and DENV-4 contained the first 300 and 272 amino acids of their respective NS5 proteins. The preparations of NS5 and MTase proteins were reported previously [15], [37].

The second assay measured [^3^H-methyl] incorporation into non-biotinylated RNA substrates. The reaction (20 µl) contained 50 mM Tris-HCl (pH 9.0), 2 µg MTase of DENV-4, 1 µCi of [^3^H-methyl]-SAM, and 1 µg of oligo RNA, viral RNA, 18 S rRNA, or 28 S rRNA. After incubation at room temperature for 1 h, the unincorporated [^3^H]-SAM was removed by RNeasy kit (Qiagen, Valencia, CA USA) according to the manufacture's instruction. The RNA samples were then mixed with 50 µl of optiphase supermix (Perkin Elmer), and measured for [^3^H-methyl] incorporation by a MicroBeta counter.

A third assay involved LC-MS analysis of Am following treatment of polyA, 18S rRNA and 28S rRNA (1 µg) with DENV-4 MTase (2 µg) in a reaction (total volume 20 µl) containing 50 mM Tris-HCl (pH 9.0), 2 mM DTT and 50 µM SAM, with incubation at room temperature for 1.5 h. In addition, genomic RNA purified from DENV virion was directly analyzed using LC-MS (see below).

### Isolation of DENV-1 genomic RNA

For analysis of internal adenosine methylation of genomic RNA, WT and MTase E217A mutant DENV-1 virions, grown in mosquito C6/36 cells, were purified. Briefly, C6/36 were infected with DENV-1 at an MOI (multiplicity of infection) of 0.1 and incubated at 29°C for five days. Cell culture supernatants were then harvested and virus were precipitated using 8% PEG8000 (w/v) at 4°C overnight. Precipitated virus was then resuspended in NTE buffer (12 mM Tris-HCl, 120 mM NaCl, 1 mM EDTA, pH 8.0) and purified by spinning the virus through a 24% (w/v) sucrose cushion at 75,350× g for 1.5 h at 4°C. Virus pellet was resuspended into 4% (w/v) potassium tartrate in NTE buffer and centrifuged at 149,008× g for 2 h at 4°C. Virus was further purified in a 10–30% potassium tartrate gradient by spinning at 126,444× g for 2 h at 4°C. Virus band of WT or E217A mutant was collected and concentrated using Millipore Amicon Ultra 100 K MWCO (Molecular Weight Cutoff). Virus samples were analyzed on a 15% SDS-PAGE stained with Coomassie brilliant blue to visualize viral capsid, membrane (pre-membrane), and envelope proteins. The amount of purified WT and E217A mutant virus were similar. Genomic RNAs were extracted from the purified virions using trizol (Invitrogen), quantified using NanoDrop, and subjected to enzymatic hydrolysis as described below.

### LC-MS analysis and quantification of Am in DENV genomic RNA and RNA treated with MTase *in vitro*

Identification and quantification of Am in samples of MTase-treated polyA, and 18S and 28S rRNA, and in DENV genomic RNA was achieved by analysis of RNA-derived ribonucleosides by HPLC-coupled mass spectrometry. For all analyses, samples of RNA (1–3 µg) were treated with 1 U/µl nuclease P1, 2.5 mM deferoxamine mesylate (antioxidant), 10 ng/ml coformycin (adenosine deaminase inhibitor), 50 µg/ml tetrahydrouridine (cytidine deaminase inhibitor), and 0.5 mM butylated hydroxytoluene (antioxidant) at 37°C. After 3 h, alkaline phosphatase and phosphodiesterase I were added to a final concentration of 0.1 U/ml. The sample was incubated at 37°C overnight, followed by removal of enzymes by filtration through a 10,000 kDa-molecular weight cut-off Amicon spin filter. The resulting filtrate was lyophilized prior to mass spectrometric analysis.

For identification of Am in polyA treated with MTase, the lyophilized hydrolysis products were dissolved in deionized water and analyzed by HPLC-coupled, electrospray ionization (ESI) quadrupole time-of-flight mass spectrometry (LC-QTOF). To resolve ribonucleosides, the digested sample (5 µl) was loaded onto a Thermo Hypersil aQ column (100×2.1 mm, 1.9 µm particle size) at 25°C and eluted at a flow rate of 50 µl/min with an acetonitrile gradient using the following mobile phases: Solvent A: 0.1% formic acid in 10 mM ammonium acetate (pH 7.5); and Solvent B: 0.1% formic acid in acetonitrile. The percentage of Solvent B was as follows: 0–5 min, 0.5%; 5–14 min, 5%; 14–19 min, 5%; 19–31 min, 23%; 31–34 min, 23%; 34–49 min, 83%; 49–54 min, 83%; 54–60 min, 0.5%; 60–70 min, 0.5%. The eluent was analyzed on an Agilent QTOF 6520 mass spectrometer with an ESI source operated in positive ion mode and the mass spectrometer was operated in ion scanning mode (*m/z* 100–1000) with the following parameters: gas temperature, 350°C; drying gas, 10 l/min; fragmentor voltage, 100 V; skimmer voltage, 65 V; and capillary voltage, 3500 V. Data processing was performed using MassHunter Workstation software version B.03.01 (Agilent Technologies, Santa Clara CA USA). The retention times and exact molecular weights of Am and other methylated ribonucleoside species were compared to chemical standards (C_m_, U_m_, G_m_, m^3^C/m^5^C, m^6^_2_A, m^1^A/m^6^A, and m^7^G).

To quantify Am, samples of RNA (MTase-treated 18S and 28S rRNA, and DENV genomic RNAs) were hydrolyzed to ribonucleosides, as described above, followed by resolution of the ribonucleosides on a Thermo Hypersil aQ column (100×2.1 mm, 1.9 µm particle size) at 25°C at a flow rate of 0.3 ml/min with an acetonitrile gradient in 0.1% (v/v) formic acid in water as follows: 0–1.5 min, 0%; 1.5–2.7 min, 6%; 2.7–4.4 min, 17.8%; 4.4–5.0 min, 18%; 5.0–5.5 min, 18%; 5.5–8.1 min, 30%; 8.1–9.3 min, 40%; 9.3–10.2 min, 65%; 10.2–11.1 min, 95%; 11.1–12.0 min, 95%; 12.0–12.9 min, 0%; 12.9–15.0 min, 0%. The HPLC column was coupled to a triple quadrupole mass spectrometer (LC-MS/MS) operated in positive ion, multiple reaction monitoring (MRM) mode for the Am molecular transition of *m/z* 282→136. Voltages and source gas parameters were as follows: gas temperature, 300°C; sheath gas temperature, 325°C; gas flow, 8 l/min; nebulizer, 40 psi; and capillary voltage, 4000 V. Quantification of Am and adenosine in the MTase-treated and DENV viral RNA samples was achieved by integrating the extracted ion chromatographic peaks for molecular transitions *m/z* 282→136 and *m/z* 268→136, respectively, followed by interpolation from linear external calibration curves prepared with Am (0.5–10 nM) or adenosine (0.1–10 µM) dissolved in the hydrolysis buffer as a matrix control. The number of Am per viral genome was calculated by multiplying the measured value of Am per adenosine by the number adenosines in the 10,735 ribonucleotide DENV-1 genome (2861; NCBI Genome Database; http://www.ncbi.nlm.nih.gov/genome?term=dengue%20virus%201).

### Replicon assay

*Renilla* luciferase (Rluc)-reporter replicons of DENV-1 (Western Pacific 74 strain; GenBank accession U88535) [45] and WNV (New York strain 3356) [36] were used to examine the effect of internal methylation on viral translation and RNA synthesis. Replicon RNAs were *in vitro* transcribed using mMESSAGE mMACHINE kits (Applied Biosystems). The 5′ end of replicon or genome-length RNA was treated with vaccinia virus capping enzyme and VP39 to generate m^7^GpppAm-RNA following the manufacturer's protocols (Epicetre). The reactions were extracted with phenol∶chloroform and precipitated using ethanol. The resulting replicon RNAs (4 µg) were treated with 2 µg of WT and mutant WNV or DENV-1 MTases in the presence of 50 µM SAM in the methylation buffer described above. After incubation at room temperature for 1 h, the reaction mixtures were directly electroporated to 8×10^6^ BHK-21 cells [46]. The electroporated cells were resuspended in 20 ml of DMEM medium with 10% FCS. 0.5∼1.0 ml of cells were seeded onto a 12-well plate (2∼4×10^5^ cells per well), and assayed for luciferase activities at indicated time points. The luciferase assay was performed as reported previously [47].

Besides measuring luciferase activity, we also quantified intracellular viral RNA at various time points after electroporation. For each time point, total cellular RNA was extracted using RNeasy kit (Qiagen). The extracted RNAs (3 µg) were subjected to standard RT-PCR quantification using one primer pair targeting viral NS5 gene (forward primer 5′-TGAAGCTAAGGTGCTTGAGC-3′ and reverse primer 5′-AGCCACATCTGGGCATAAGA-3′) and another primer pair targeting housekeeping gene actin (forward primer 5′-AGAGGGAAATTGTGCGTGAC-3′ and reverse primer 5′-CAATGGTGA TGACCTGGCCA-3′) The RT-PCR reactions were performed using SuperScript III one-step RT-PCR kit (Invitrogen), and the products were analyzed on a 1% agarose gel.

### Replication assay using DENV-1 genome-length RNA

Genome-length RNA of DENV-1was *in vitro* transcribed from a full-length cDNA plasmid linearized by *SacII* [48]. Using the same protocol for replicon experiment (described above), the genome-length RNA containing a 5′ m^7^GpppAm cap (2 µg) was treated with WT and mutant DENV-1 MTases. The internally methylated genome-length RNAs were electroporated into BHK-21 cells [46]. The transfected cells were resuspended in 20 ml of DMEM medium, and subjected to virus production and specific infectivity assays. For virus production assay, 18 ml of the resuspended cells plus 10 ml of medium were cultured in a T-175 flask. Viral titers of culture fluids collected on day 1–7 post-transfection (p.t.) were determined using a single-layer plaque assay [37]. For specific infectivity assay, a series of 1∶10 dilutions of the transfected cells were prepared using DMEM medium. One ml of cell suspension at each dilution was seeded onto confluent BHK-21 cell in six-well plates (The plates were seeded with 5×10^5^ BHK-21 at 16–24 h before the assay day). After incubating the plates for 6 h (to allow the transfected cell to attach to the monolayer of BHK-21 cells), culture medium was aspirated and replaced with an overlayer medium (RPMI 1640, 2% FBS, 1% penicillin-streptomycin, and 0.8% methylcellulose). The plates were incubated at 37°C with 5% CO_2_ for 5 days. The cells were then fixed with 10% formaldehyde for 20 min at room temperature, rinsed with tap water, and stained with 1% crystal violet for 5 min. The plates were again rinsed with tap water (to remove staining) and visually counted for plaques. The specific infectivity was calculated as the number of infectious virus upon transfection of 1 µg of genome-length RNA.

### RNA elongation assay

The sequences of RNA template and primer are shown in Figure 7D. The two RNAs (12.5 µM) were annealed in 50 mM Tris-HCl (pH 7.0) and 100 mM NaCl by heating at 95°C for 3 min followed by cooling to room temperature (23°C). The RNA elongation reaction (25 µl) contained 50 mM Tris-HCl (pH 7.0), 50 mM NaCl, 5 mM MgCl_2_, 2 mM MnCl_2_, 0.25 µM annealed RNA template/primer, 1 µM cold UTP, 1 µM ^3^H-UTP, 4 mM DTT, and 50 nM full-length DENV-4 NS5. After incubating the reaction at room temperature for 1 h, the reactions were terminated by addition of an equal volume of 2× stop solution (containing 100 mg of SPA beads coated with streptavidin in 50 mM This-HCl [pH 7.0], 40 mM EDTA, and 150 mM NaCl). The 96-well plate was agitated at room temperature for 15 min, and measured for ^3^H-UTP incorporation as described above.
